# Liver hypertrophy techniques: a position paper from the Italian Group of Regenerative and Occlusive Worldwide-used techniques of hepatic Hypertrophy (I GROWtoH)

**DOI:** 10.1007/s13304-025-02364-1

**Published:** 2025-08-26

**Authors:** Matteo Serenari, Francesca Ratti, Mohammed Abu Hilal, Francesco Ardito, Giammauro Berardi, Ugo Boggi, Alberta Cappelli, Matteo Cescon, Umberto Cillo, Alessandro Cucchetti, Luciano De Carlis, Francesco De Cobelli, Fabrizio Di Benedetto, Giorgio Ercolani, Giuseppe Maria Ettorre, Massimo Fedi, Alessandro Ferrero, Felice Giuliante, Gian Luca Grazi, Enrico Gringeri, Salvatore Gruttadauria, Francesco Izzo, Marcello Maestri, Paolo Magistri, Marco Massani, Vincenzo Mazzaferro, Riccardo Memeo, Federico Mocchegiani, Cristina Mosconi, Damiano Patrono, Matteo Ravaioli, Fabrizio Romano, Gianluca Rompianesi, Nadia Russolillo, Andrea Ruzzenente, Carlo Sposito, Roberto Troisi, Giovanni Vennarecci, Luca Viganò, Marco Vivarelli, Giacomo Zanus, Pedro M. Baptista, Karl Oldhafer, Erik Schadde, Luca Aldrighetti, Elio Jovine

**Affiliations:** 1 Hepato-Biliary Surgery and Transplant Unit, IRCCS Azienda Ospedaliero-Universitaria Di Bologna, Via Albertoni 15, 40138 Bologna, Italy; 2 Department of Medical and Surgical Sciences, Alma Mater Studiorum, University of Bologna, Bologna, Italy; 3 Hepatobiliary Surgery Division, IRCCS San Raffaele Scientific Institute, Milan, Italy; 4 Vita-Salute San Raffaele University, Milan, Italy; 5 Department of HPB Surgery, University Hospital Southampton, Southampton, UK; 6 Hepatobiliary Surgery Unit, Fondazione Policlinico Universitario A. Gemelli IRCSS, Università Cattolica del Sacro Cuore, Rome, Italy; 7 Department of Surgery, San Camillo Forlanini Hospital, Rome, Italy; 8 Department of Surgery, University Hospital of Pisa, Pisa, Italy; 9 Department of Radiology, IRCCS Azienda Ospedaliero-Universitaria Di Bologna, Bologna, Italy; 10 General Surgery 2—Hepato-Pancreato-Biliary Surgery and Liver Transplantation Unit, Padua University Hospital, Padua, Italy; 11 General and Oncology Surgery, Morgagni-Pierantoni Hospital, Ausl Romagna, Forlì, Italy; 12 Department of General Surgery and Transplantation, School of Medicine, Niguarda Hospital—University of Milano-Bicocca, Milan, Italy; 13 Department of Radiology, IRCCS San Raffaele Scientific Institute, Milan, Italy; 14 Hepato-Pancreato-Biliary Surgery and Liver Transplantation Unit, University of Modena and Reggio Emilia, Modena, Italy; 15 Hepatobiliary Surgery Unit, USL Toscana Centro—San Jacopo Hospital, Pistoia, Italy; 16 Department of Surgery, Mauriziano Hospital, Turin, Italy; 17 HepatobiliaryPancreatic Surgery, Department of Experimental and Clinical Medicine, University of Florence, Florence, Italy; 18 Department Abdominal Center, UPMC (University of Pittsburgh Medical Center), 90127 Palermo, Italy; 19 Department of Surgery and Medical and Surgical Specialties, University of Catania, 95124 Catania, Italy; 20 Division of Surgical Oncology, Hepatobiliary Unit, Istituto Nazionale Tumori IRCCS Fondazione “G. Pascale”, Napoli, Italy; 21 Division of General Surgery 1, Department of Surgery, Fondazione IRCCS Policlinico San Matteo, 27100 Pavia, Italy; 22 Regional Center for HPB Surgery, Regional Hospital of Treviso, Treviso, Italy; 23 Department of Surgery, Division of HPB Surgery and Liver Transplantation, Fondazione IRCCS Istituto Nazionale Tumori Di Milano, Milan, Italy; 24 Department of Oncology and Hemato-Oncology, University of Milan, Milan, Italy; 25 Division of Hepato-Pancreato-Biliary Surgery, “F. Miulli” General Hospital, Acquaviva Delle Fonti, Bari, Italy; 26 HPB Surgery and Transplantation Unit, Department of Clinical and Experimental Medicine, Polytechnic University of Marche, Ancona, Italy; 27 General Surgery 2U-Liver Transplant Unit, Department of Surgical Sciences, Azienda Ospedaliero Universitaria Città Della Salute E Della Scienza Di Torino, Università Di Torino, Turin, Italy; 28 School of Medicine and Surgery, Hepatobiliary Surgery, University of Milan Bicocca, IRCCS San Gerardo, Monza, Italy; 29 Division of HPB, Minimally Invasive and Robotic Surgery, Transplantation Service, Federico II University Hospital, Naples, Italy; 30 Division of General and Hepatobiliary Surgery, G.B, University of Verona, Rossi University Hospital, Verona, Italy; 31 Department of Hepatobiliary and Liver Transplantation Surgery, A.O.R.N. Cardarelli, Naples, Italy; 32 Department of Minimally Invasive General & Oncologic Surgery, Hepatobiliary Unit, Humanitas Gavazzeni University Hospital, Bergamo, Italy; 33 Department of Biomedical Sciences, Humanitas University, Milan, Pieve Emanuele Italy; 34 Department of Surgical, Oncological and Gastroenterological Science (DISCOG), University of Padua, Padua, Italy; 35 Hepatobiliary and Pancreatic Surgery Unit, Treviso Hospital, Treviso, Italy; 36 Laboratory of Organ Bioengineering and Regenerative Medicine, Health Research Institute of Aragon (IIS Aragon), Saragossa, Spain; 37 Biomedical Engineering Department, Carlos III of Madrid University, Madrid, Spain; 38 ARAID Foundation, Saragossa, Spain; 39 Biomedical Research Networking Center in Hepatic and Digestive Diseases (CIBERehd), Madrid, Spain; 40 Department of Surgery, Division of Hepatobiliary and Pancreatic Surgery, Asklepios Hospital Barmbek, Hamburg, Germany; 41 Department of Surgery, Rush University Chicago, Chicago, IL USA; 42 Chirurgisches Zentrum Zürich (CZZ), Klinik Hirslanden Zurich, Zurich, Switzerland; 43 VISCERA Lucerne, Hirslanden St. Anna, Lucerne, Switzerland; 44 Department of General Surgery, IRCCS Azienda Ospedaliero-Universitaria Di Bologna, Bologna, Italy

**Keywords:** Liver hypertrophy, Portal vein embolization, Liver function, Liver volume

## Abstract

In candidates for hepatectomy, different techniques to induce liver hypertrophy and modulate the future liver remnant are available. However, their use in specific clinical scenarios is highly heterogeneous and there is no consensus about minimal safety standards needed to incorporate these strategies into routine clinical practice. The aim of this position paper was to summarize newly available evidence in the field and compare medical practice among different hepatobiliary surgical units to evaluate the transformative potential of liver hypertrophy techniques in surgical oncology. This paper sets the stage for a future structured consensus on the application of liver hypertrophy techniques before hepatectomy.

## Introduction

Post-hepatectomy liver failure (PHLF) significantly increases both morbidity and mortality after liver surgery. [1] To prevent PHLF, different techniques have been developed to induce liver hypertrophy and optimize future liver remnant (FLR), including portal vein ligation (PVL), portal vein embolization (PVE), and two-stage hepatectomy (TSH), associating liver partition and portal vein ligation for staged hepatectomy (ALPPS) and double vein embolization (DVE)/liver venous deprivation (LVD) [2]. The multiplicity of these approaches provides evidence of specific limitations of each technique and the desire of clinicians to reduce the risk of dropout from curative surgery at minimal risk to the patients’ well-being.

Since so many options are available, choosing the right technique for the right patient may be challenging. One has to consider the oncologic indication of the intended liver surgery, the patient performance status and comorbidities, the underlying liver function and volume, and the expected outcomes in terms of FLR hypertrophy, drop-out and peri-procedural/operative morbidity [3].

However, the application of hypertrophy techniques across different hepato-pancreatic-biliary (HPB) surgical units varies considerably, being influenced by personal preferences, experience, and local resource availability. Literature on the subject is heterogeneous and there is limited consensus on which is the best technique for specific clinical scenarios or the minimum safety standards required for their implementation in routine practice. A recent survey disseminated within the Italian HPB community to investigate availability and use of techniques to induce FLR hypertrophy highlighted the perceived importance of these approaches to enhance resectability rate, but also empirically supported an unusual variability in indications, protocols, and minimum volumetric cutoff values across different centers [4].

## Materials and methods

A core panel comprising 41 Italian experts (surgeons and radiologists) belonging to the I GROWtoH community was identified by the Organizing Committee (LA, EJ, FR, MS). The organizing committee shared a tentative list of topics and clinical questions with the expert panel until a final document was obtained. Within each topic, a subgroup of clinical questions was assigned to each expert. The expert team was responsible for identifying relevant evidence through a comprehensive literature search on the assigned topic (using PubMed, MEDLINE, EMBASE, Scopus, Cochrane Library, and Google Scholar electronic databases). The output of the work from experts was presented during a dedicated meeting held in Bologna (Italy) in January 2024. During meeting, an external review board was invited (PMB, KO, ES) to assess the process and promote critical discussions. Based on the literature search and the quality of evidence, recommendations were drafted and proposed during a second “I GROWtoH meeting”, held during the AICEP congress in Padua (Italy) in April 2024. Each proposed recommendation was revised until a ≥ 90% agreement was reached among all members of the working panel. The revised final draft was externally reviewed by the external review board.

The final report is divided into two parts: 1. Techniques to induce liver hypertrophy; 2. Candidates for surgery.

## Techniques to induce liver hypertrophy

### Portal vein ligation (PVL)

Portal vein ligation, first proposed by Honjo and colleagues in 1975 [5], involves the ligation of the portal vein branch that supplies the part of the liver to be resected (usually the right portal vein), thereby redirecting the blood flow to the remaining segments and stimulating FLR hypertrophy. PVL can be performed by laparotomy or by a minimally invasive approach. [6] According to a recent meta-analysis, mean FLR increase induced by PVL was 38.5% (95% CI 29.2–47.7) [7] and a waiting period of 4–6 weeks is observed before the second stage is completed. Since the formation of intrahepatic collaterals from segment 4 to segments 5 and 8 may limit the hypertrophic response, [8] alcohol injection distal to PVL has been advocated to reduce the recanalization of the occluded portal vein and increase FLR hypertrophy [9].

#### What is the current role of PVL?

Feasibility and reproducibility of PVL have been reported in both open and minimally invasive approach. [6] PVL is usually employed in two-step procedures (e.g., two-stage hepatectomy or associated liver partition and portal vein ligation for staged hepatectomy, ALPPS) where a first surgical step is required usually in case of bilobar colorectal liver metastases (CRLM). [10]

##### Expert panel recommendations

- PVL should be reserved for cases where a first surgical step is required (e.g., clearance of the FLR) such as in two-stage hepatectomies or ALPPS;
- PVL can be combined with parenchymal splitting or alcohol injection to limit the development of intrahepatic collaterals, thereby increasing the rate of FLR hypertrophy.

### Portal vein embolization (PVE)

Portal vein embolization (PVE) was first described by Makuuchi et al. in 1984 as a preoperative method to induce FLR hypertrophy. [11] This technique involves the occlusion of the portal flow to the hemiliver to be removed using various embolizing agents, such as polyvinyl alcohol particles, coils, gelatin sponge, *n*-butyl cyanoacrylate (NBCA), lipiodol, and fibrin glue. PVE may be performed via a transileocolic approach (TIPE) during laparotomy or by a percutaneous transhepatic (PTPE) [12] or transsplenic approach [13] using ultrasonic guidance under local anesthesia. The TIPE approach requires surgical cannulation of the ileocolic vein and is usually performed as part of a two-stage resection in hybrid operating rooms. In the transhepatic approach, the portal branch to be occluded is accessed via either ipsilateral (tumor side) or contralateral puncture. The main advantage of transhepatic ipsilateral approach is a lower risk of FLR damage. However, the acute angulation of the right portal vein may result in the displacement of embolization materials after catheter removal. [14]

#### Is PVE superior to PVL?

According to two recent meta-analyses [7, 15], PVE is not superior to PVL in terms of FLR hypertrophy. Morbidity and mortality did not differ significantly between the two procedures. However, PVL usually requires dissection of the hepatic hilum, and adhesions resulting from this procedure may increase the complexity of the second operation.

#### What is the best radiological approach to PVE (access, embolizing agents)?

According to the meta-analysis of Abulkhir et al. [16], PTPE and TIPE do not significantly differ in terms of major complications although PTPE was associated with greater FLR increase, whereas TIPE had fewer drop-outs between stages. Similarly, no significant differences in complication rates were observed between contralateral and ipsilateral PTPE approaches.

Regarding embolizing agents, the use of NBCA appears to result in greater hypertrophy compared to microparticles [17] or polyvinyl alcohol. [18]

#### Should embolization of segment 4 be routinely performed in candidates for right trisectionectomy?

Embolization of the S4 branches of the portal vein has been shown to result in a significantly greater increase in FLR volume compared to right PVE alone in patients scheduled for right trisectionectomy. [18] However, this approach may increase the risk of unintended occlusion of the left portal vein and, consequently, of the FLR, limiting its widespread adoption. Nonetheless, there is no clear evidence in the literature that S4 embolization significantly increases PVE-associated complication rates.[19, 20]

#### What outcomes can be expected after PVE (hypertrophy rate, drop-out, peri-procedural morbidity)?

According to a recent systematic review, approximately 20% of patients did not complete the second stage of the procedure. Among the reported causes of drop-out, tumor progression was the most frequent (14.2% of cases). The mean interval between PVE and hepatectomy was 36 days (range 21–84), with a mean FLR volume increase of 37.9 ± 0.1% (range 20.5–69.4%). The procedure-related mortality rate was 0.1%, and the major complication rate was 0.4%. [12]

##### Expert panel recommendations

- PVE is the recommended standard hypertrophy technique in patients requiring FLR augmentation.
- The choice of approach (access site and/or embolic agent) should be at the discretion of the interventional radiologist;
- The use of *n*-butyl cyanoacrylate glue (NBCA) appears to result in greater hypertrophy than other embolic agents and is therefore recommended;
- Embolization of S4 is cautiously recommended only in patients undergoing right trisectionectomy and should be performed based on interventional radiology expertise as it may increase the risk of accidental FLR occlusion.

### Double vein embolization (DVE)/Liver venous deprivation (LVD)

Double vein embolization (DVE) is a technique which selectively occludes both the hepatic vein and the portal vein, either simultaneously or sequentially, to enhance FLR hypertrophy. By increasing portal flow to the non-embolized segments and minimizing intrahepatic collateral formation, DVE stimulates a more robust regenerative response. First introduced by Hwang et al. in 2009 [21] as a sequential rescue strategy for patients with insufficient FLR (≤40% of total liver volume) after PVE alone, it was further developed by Guiu et al. in 2016[22] who proposed simultaneous embolization of both portal and hepatic veins in patients with inadequate FLR volume. Typically, DVE consists of right PVE combined with right hepatic vein embolization (HVE); middle hepatic vein embolization may also be considered in patients undergoing right trisectionectomy [23] For HVE, an Amplatzer plug is placed approximately 10–15 mm from the hepato-caval confluence. When glue is injected distal to the plug, the procedure is referred to as liver venous deprivation (LVD). [14] Contraindications to DVE/LVD include ipsilateral tumor thrombus and clinically relevant portal hypertension. [3]

#### Is DVE/LVD superior to PVE?

According to a recent meta-analysis including eight comparative studies and six retrospective DVE/LVD case series [24], this study was associated with a higher resectability rate and greater FLR hypertrophy compared to PVE without increasing procedural morbidity. These findings suggest that DVE/LVD may serve as a primary hypertrophy strategy, particularly in patients with very low baseline FLR volume or function. [25] However, definitive conclusions await results from two ongoing prospective multicenter randomized controlled trials comparing DVE/LVD to PVE. [26, 27]

#### What is the best radiological approach to DVE/LVD?

Both transhepatic and transjugular approaches have been employed for DVE/LVD with similar safety profiles and comparable outcomes in terms of hypertrophy and resectability. [28] The transjugular approach offers the advantage of fewer hepatic punctures, potentially reducing intrahepatic trauma. Conversely, the transhepatic route allows distal glue injection, which may help prevent the formation of intrahepatic venous anastomoses.

#### What outcomes can be expected after DVE/LVD (hypertrophy rate, drop-out, periprocedural morbidity)?

Systematic reviews and meta-analyses have reported FLR volume increases ranging from 36 to 67% within a median interval of 17–31 days. Resectability rates vary between 67% and 100%. In single-arm studies involving 175 patients, 146 (84%) proceeded to successful liver resection. The main reason for drop-out was disease progression (11.8%), while only 1.4% failed to achieve sufficient FLR hypertrophy. As shown in a recent report by Maina et al. [29], adhesions at the hepatic vein confluence and/or at the hepatic hilum or Amplatzer plug misplacement in the right hepatocaval confluence after DVE/LVD may both potentially increase the risk of intraoperative vascular injury.

##### Expert panel recommendations

- DVE/LVD is the recommended first rescue procedure in case of insufficient hypertrophy following PVE;
- In the near future, the use of DVE/LVD as a primary hypertrophic technique is expected to significantly increase in patients who require intensive FLR augmentation. However, results from the ongoing RCTs are not yet available and no definitive recommendations can be made at present time;
- The choice of performing a simultaneous or sequential approach as well as choosing between DVE or LVD should be at the discretion of the interventional radiologist.

### Two-stage hepatectomy (TSH)

Two-stage hepatectomy (TSH) is a feasible and reproducible surgical technique to increase the chances of resectability in patients with bilobar CRLM. The most frequently adopted approach includes clearance of the left lateral sector or left hemiliver (by surgical resection or thermal ablation) as described by Adam et al [30]. Either PVL [31] or PVE [32] may be performed to induce FLR hypertrophy, prior to subsequent right trisectionectomy/hepatectomy.

#### What is the current role of TSH?

TSH is still a widely used technique in case of bilobar liver metastases when the clearance of small FLR is required. When compared with Associating Liver Partition and Portal vein ligation for Staged hepatectomy (ALPPS), there is still much debate on which is the best technique in terms of both short- and long-term-outcomes, especially considering the only RCT published in this regard and the available meta-analyses which, however, comprise only retrospective series [33–36].

A few case series of laparoscopic TSH have also been described in recent literatures [37], [38], [39]. No significant differences after propensity score matching were found in a recent study between laparoscopic and open approach [40], but the minimally invasive first stage seemed to limit adhesions in the second stage. [41]

#### What outcomes can be expected after TSH (hypertrophy rate, drop-out, peri-procedural morbidity)?

According to the systematic review of Lam et al. [42], postoperative morbidity and mortality after stage 1 were 17% (range: 0–26) and 0.5%, respectively. The drop-out rate was 23% with interval disease progression being the main reason for non-completion of the second stage (88%), followed by inadequate FLR volume (4%). Postoperative morbidity and mortality after stage 2 were 40% (range: 20–56) and 3%, respectively. All postoperative deaths were related to PHLF.

##### Expert panel recommendations

- TSH is still a widely adopted and validated technique to treat patients with bilateral CRLM and can be safely performed in patients with bilobar CRLM and low FLR;
- A minimally invasive approach to the first stage, if potentially feasible and safe, is recommended.

#### Associating liver partitioning and portal vein ligation for staged hepatectomy (ALPPS)

Associating Liver Partitioning and portal vein Ligation for Staged hepatectomy (ALPPS) was first named “in situ-split hepatectomy” and described by Prof. Hans Schlitt in 2012. [43] Despite the high mortality reported in the early experience with the ALPPS procedure, [44], [45] more favorable results have been reported in recent years. [46] Improvements in the original technique (PVL and total splitting of the liver) such as mini-ALPPS (i.e., PVE and partial splitting), the minimally invasive approach to stage 1 [46] and the use of functional tests to reduce the risk of PHLF have been described over the years. [47] Both PVE and PVL can be performed and currently no evidence supports the use of one technique over the other. However, PVE seems to limit surgical adhesions at the hepatoduodenal ligament, facilitating second stage. Similarly, both total and partial splitting can be performed possibly providing similar results in terms of FLR hypertrophy and most likely, a lower morbidity rate in partial ALPPS. [48] This effect can be also explained by the higher rate of minimally invasive approaches associated with partial parenchymal splitting. The combination of DVE/LVD and liver splitting has recently been described [49], representing a possible strategy to obtain further and more rapid increase of FLR volume.

#### What is the current role of ALPPS?

ALPPS represents nearly 2% of all liver resections according to a recent multicenter Italian report [46]. In general, ALPPS should be reserved to patients with very small FLR, borderline resectable tumors approaching the FLR or failed PVE/DVE/LVD (namely “rescue ALPPS”) [50], [51].

When compared with TSH for CRLM, rates of complications were similar between these two techniques according to a recent RCT. [33] However, when looking at two other recent meta-analyses, major complications were higher in the ALPPS group while 90-day mortality was similar between the two groups. [34], [35] Despite a higher rate of resectability in ALPPS which may explain the higher OS in the ALPPS group as shown in the LIGRO trial, [36] the published meta-analyses in this regard did not show any survival advantage of ALPPS over TSH. [34], [35]

In HCC, another peculiar indication may be represented by the presence of portal vein thrombosis [52] which is usually a major contraindication to PVE. Conversely, high mortality associated with ALPPS in biliary tumors [53] likely constitutes an absolute contraindication to its use in this setting. Published risk scores may help in selecting these patients, thereby avoiding the futility of the procedure. [54], [55], [56]

#### What outcomes can be expected after ALPPS (hypertrophy rate, drop-out, peri-procedural morbidity)?

Median FLR hypertrophy prior to the second stage operation after ALPPS for primary liver malignancies was 54%, ranging from 6.7 to 133% in a recent systematic review [57] even though heterogeneity among the included studies must be considered (interval days, quality of underlying liver parenchyma, type of splitting). After the initial adoption of this technique, morbidity decreased from 52.4% in 2012 to 28.5% in 2020 according to the data from the ALPPS Italian registry. Mortality remains high with variable rates according to the indication for surgery: [45] in particular, mortality in ALPPS for perihilar cholangiocarcinoma (pCCA) was reported to be as high as 48%.[53] In a more recent single-arm meta-analysis, the estimated mortality for pCCA was 22%, still limiting the use of this technique in these patients. [58] Resectability after ALPPS was reported to be higher when compared with other hypertrophy techniques. More specifically, the feasibility rate (percentage of patients reaching stage 2) in a meta-analysis comparing ALPPS and conventional staged hepatectomies was 97%. [59]

##### Expert panel recommendations

- ALPPS can be justified in case of very small FLR, failure of previous hypertrophy techniques or borderline resectable tumors near the FLR when a high risk of drop-out is expected;
- ALPPS can be safely performed in CRLM setting provided that careful selection of the patient is carried out;
- ALPPS should be avoided for biliary tumors (particularly pCCA) due to the associated high risk of mortality;
- Although PVE or PVL both can be used interchangeably to occlude the portal vein as can partial or total parenchymal splitting, PVE and partial splitting are recommended;
- A minimally invasive approach to the first stage, if potentially feasible and safe, is recommended.

#### Trans-arterial radioembolization (TARE)

Trans-arterial radioembolization (TARE) has emerged as an effective locoregional therapy for primary and secondary hepatic tumors in which trans-arterially injected yttrium-90 (⁹⁰Y)-loaded microspheres act as a vehicle for delivery and administration of radiation with a low microembolic effect. TARE can be performed selectively on the tumor to be treated, targeting a precise segment or even an entire liver lobe. Lobar radioembolization, also known as radiation lobectomy, was first described in 2009 as an incidental imaging finding of ipsilateral hepatic lobar atrophy and contralateral lobar hypertrophy after lobar TARE. [60] When tumor biology is tested by not resecting in short intervals or increasing waiting time in the presence of effective chemotherapy, TARE may be helpful.

#### Can radioembolization have a role in inducing FLR hypertrophy prior to liver resection?

According to a recent systematic review [61], TARE resulted in contralateral liver hypertrophy ranging from 26 to 47% over an interval ranging from 44 days to 9 months. However, the studies were retrospective in nature and heterogeneous, with substantial variations in terms of the disease treated, underlying liver disease, ^90^Y dosage and delivery, number of treatment sessions and timing of hypertrophy measurement.

##### Expert panel recommendations

- TARE can be used as a loco-regional treatment to achieve downstaging of unresectable or borderline resectable tumors when a test of time/biology is intended, but it is not recommended as a primary method to induce FLR hypertrophy.

## Candidates for surgery

### Pre-operative assessment

Liver volumetry was first described in 1979 by Heymsfield et al. [62], but it was only in the mid-1980s that it began to be used to estimate the volume of the FLR in donor hepatectomy [63] and prior to oncological liver surgery. [64] Since then, hepatic volumetry has become a standard part of preoperative assessment before any major hepatectomy. Liver volumetry can be performed manually using either CT or magnetic resonance imaging (MRI) [65] However, as manual volumetry is time-consuming, semiautomated and fully automated methods have been developed. No significant differences in accuracy have been reported between manual and semiautomated methods. [66] Fully automated volumetry is less time-consuming [67], but its advantage over semi-automated volumetry remains to be demonstrated.

#### Is hepatic volumetry still the gold standard before liver hypertrophy techniques and prior to surgery?

Although no randomized studies have directly compared methods to assess preoperative FLR adequacy, volumetry is generally considered as the gold standard to assess the risk of PHLF. However, as outlined in the recent E-AHPBA–ESSO–ESSR Innsbruck consensus guidelines [68], the role of volumetry may be limited in cases involving rapid FLR hypertrophy techniques (e.g., ALPPS) or in the presence of underlying liver disease, such as steatosis, cirrhosis, prior chemotherapy, and cholestasis. In these contexts, a functional assessment in addition to volumetry is recommended although two-stage hepatectomies were not included in the consensus algorithm [68]. The correlation between liver volume and function has been shown to be lower in two-stage hepatectomies compared to one-stage procedures, supporting the use of functional tests in all these cases [69]. In patients undergoing PVE alone, a routine use of a functional test such as hepatobiliary scintigraphy (HBS) may help avoid unnecessary additional hypertrophy procedures [70]. Interestingly, in a single-center study by Guiu et al., functional gain appeared to exceed volumetric growth following DVE/LVD much more than after PVE [71]. Nevertheless, only a few prospective comparative studies between volume and function assessment have been published so far.

##### Expert panel recommendations

- Remnant liver volume remains the gold standard for assessing the risk of PHLF before major hepatectomy, but the use of imaging-based liver function tests is recommended in conjunction with volumetric analysis.

#### Which is the recommended formula to assess liver volumetry?

According to a recent multicenter study [72] reviewing all available formulas to estimate total liver volume (eTLV), the two most accurate ones were those by Vauthey et al. based on either body surface area (BSA) (1267.28 × BSA − 794.41) or body weight (BW) (18.51 × BW + 191.8). [73] Use of these formulas helps reduce the risk of TLV overestimation and FLR underestimation, thereby balancing the need to avoid unnecessary hypertrophy procedures with the risk of PHLF. However, Vauthey’s formulas have only been validated in Western populations and may be less reliable in patients with previous hepatic resections, significant weight changes, or underlying liver disease. [74] Alternatively, measured TLV (mTLV) is calculated by manually measuring the total liver volume and subtracting the tumor volume using either CT or MRI. Ribero et al. [75] found that: mTLV underestimated eTLV in 60.1% of cases, mTLV was similar to eTLV (±2.5%) in 11.1%, and  that mTLV exceeded eTLV in 28.8% of cases. This suggests that mTLV is not only more labor-intensive but may also underestimate the risk of PHLF, making eTLV the preferred method. [75] Truant et al. [76] found that the FLR/BW (cutoff: 0.5) was more predictive of PHLF than the FLR/TLV. However, their study included only 31 patients, limiting the strength of their conclusions.

##### Expert panel recommendations

- Use of Vauthey’s formulas to estimate TLV and to calculate the FLR/TLV ratio should be preferred over other formulas.

### Functional tests

#### What is the best functional test in regenerative liver surgery before and after hypertrophy techniques?

Hypertrophy techniques often result in an inhomogeneous distribution of liver function. [77] Therefore, dynamic quantitative tests such as indocyanine green clearance, which measure global liver function, are insufficient when used alone to assess PHLF risk in patients undergoing FLR hypertrophy.

**Fig. 1:**
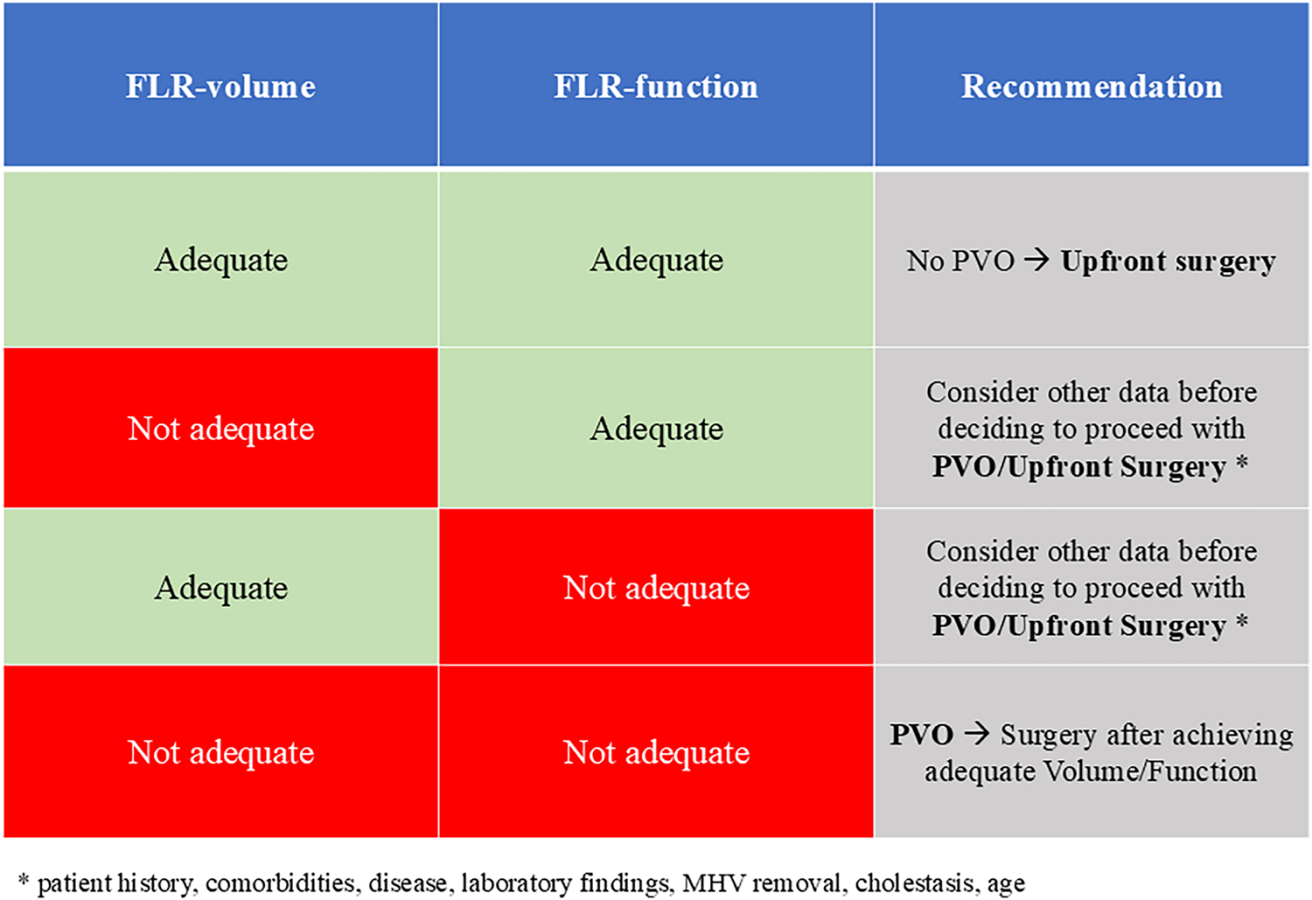
Decision-making algorithm for surgical planning based on future liver remnant (FLR) volume and function. The table presents clinical recommendations according to the combined assessment of FLR volume and function. When both parameters are adequate, upfront surgery can be performed without the need for portal vein occlusion (PVO). When either or both parameters are inadequate, further clinical data (patient history, comorbidities, laboratory findings, cholestasis, age, and middle hepatic vein [MHV] resection) should be considered before proceeding. In cases of insufficient volume and function, PVO is recommended, followed by surgery once adequate values are achieved

In this context, imaging-based tests that assess segmental or regional function—such as nuclear imaging and functional MRI—are more appropriate. Among these, hepatobiliary scintigraphy (HBS) with ^99m^Tc-mebrofenin has been validated in both one-stage and two-stage hepatectomies particularly in ALPPS [47], where standard volumetry showed poor accuracy in predicting PHLF. [78] Moreover, HBS with 99mTc-mebrofenin has also been demonstrated to potentially reduce the number of unnecessary PVE compared to liver volumetry alone [70]. Although the 2.7%/min/m^2^ cutoff remains widely used to identify patients at risk of PHLF [79], recent studies have proposed alternative cutoffs based on PHLF severity [69]. Nevertheless, existing cutoffs may result in false positives for grade B PHLF, emphasizing the need to interpret HBS results in combination with volumetry and other influencing factors (e.g., cholestasis, middle hepatic vein removal) (Fig. 1). Future standardization of imaging protocols and analysis (including radiopharmaceutical availability) has to be accomplished to allow comparisons across centers and to establish shared thresholds for predicting PHLF [80].

Gadoxetic acid-enhanced MRI is a promising imaging-based functional test, but due to a lack of clinical studies, there is no consensus yet on which indexes should be used in this context [81].

##### Expert panel recommendations

- HBS is the recommended functional test for estimating FLR function before and after hypertrophy techniques, particularly in rapid hypertrophy procedures such as ALPPS or DVE/LVD;
- HBS should be used together with liver volumetry to assess the risk of PHLF;
- Gadoxetic acid-enhanced MRI is a promising imaging-based functional test but no specific recommendations can currently be made regarding its use.

### ***Cutoffs (***Fig. 2***)***

**Fig. 2:**
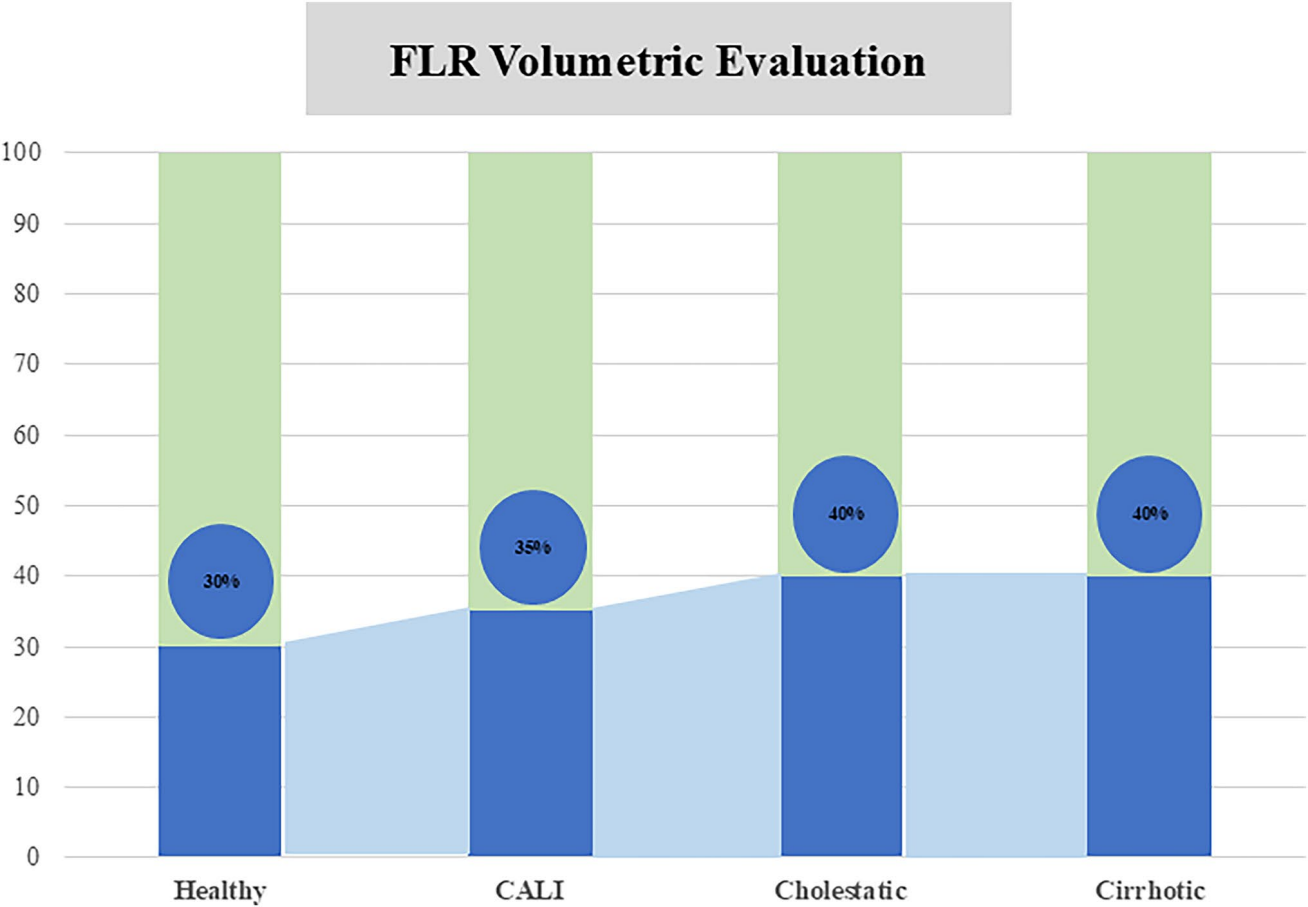
Recommended future liver remnant (FLR) volume thresholds according to underlying liver parenchyma condition. FLR cut-off values are stratified for normal liver, chemotherapy-associated liver injury (CALI), cholestatic liver disease, and cirrhosis

#### Which volume cutoff should be used in healthy livers?

Several FLR volume cutoffs for safe liver surgery in healthy livers have been reported generally ranging from 20 to 25% of TLV [82–84], [85]. Without the use of functional tests however, it is impossible to exclude an underlying liver disease. For this reason, a higher cutoff would be safer. In the living donor liver transplant (LDLT) setting, despite a lower reported PHLF rate due to stringent patient selection, post-transplant peak bilirubin and INR have been demonstrated to be significantly higher in patients with FLR <30% compared to those with FLR ≥30%, suggesting this cutoff as more appropriate in this setting [86].

#### Expert panel recommendations

- The recommended minimum FLR cutoff volume for upfront major hepatectomy in patients with a healthy liver is 25% of TLV;
- Since without the use of a functional test an underlying liver disease cannot be definitively excluded, a FLR volume ≥ 30% of TLV is recommended to minimize the risk of PHLF as in the LDLT setting.

#### Which volume cutoff should be used in steatotic livers?

Liver steatosis can be a characteristic feature of metabolic-associated steatotic liver disease (MASLD) or the consequence of prolonged exposure to chemotherapy agents such as irinotecan. [87] A recent meta-analysis revealed a significant association between the presence of moderate (≥ 30%) steatosis and the increased risk of postoperative complications and mortality [107]. Liver steatosis or more in general chemotherapy-associated liver injury (CALI) is difficult to predict preoperatively with conventional imaging such as CT. Conversely, MRI-derived proton density fat fraction (MRI-PDFF) or controlled attenuation parameter (CAP) [88, 89] appears to be able to quantify liver steatosis although no correlation with liver volume has been demonstrated so far. In a recent study by Narita et al.[90], in patients undergoing intensive preoperative chemotherapy (≥ 6 cycles of oxaliplatin or irinotecan regimen with or without targeted therapies) with a FLR/mTLV or FLR/eTLV less than 37.7% or 43.6%, respectively, FLR augmentation was recommended.

##### Expert panel recommendations

- The recommended minimum FLR cutoff volume for upfront major hepatectomy in patients who received intensive preoperative chemotherapy is 35% of TLV.

#### Which volume cutoff should be used in cholestatic livers?

Cholestasis is well-known to be associated with impaired hepatic regeneration. [91] However, its role before and after PVE is still not clear. More specifically, it is unclear which cutoff of total bilirubin should be achieved before safely proceeding with hypertrophy techniques. [92], [93, 94] Traditionally, a total bilirubin level of 2.7 mg/dl (50 mmol/L) has always been recommended before surgery or PVE [95]. Even though a FRL < 40% has frequently been used as an indication for PVE in pCCA patients [96], a recent multicenter study from Olthof et al. [97] showed that a cutoff of sFLR ≥ 45% was associated with lowest rate of PHLF (17%) in perihilar cholangiocarcinoma. For this reason, a volume is not always a reliable factor in these patients where functional tests can also be useless given that cholestasis may affect the uptake of several molecules, such as indocyanine green, mebrofenin and gadolinium [80].

#### Expert panel recommendations

- The recommended minimum FLR cutoff volume for upfront major hepatectomy in patients with cholestatic liver is 40% of TLV;
- Although a bilirubin less than 2.7 mg/dl is suggested before PVE, no definitive recommendations can be made on the optimal pre-procedural level;
- Low (≤ 2.7 mg/dl) total bilirubin levels are recommended when using liver functional tests (e.g., hepatobiliary scintigraphy).

#### Which volume cutoff should be used in cirrhotic livers?

Cirrhosis is often associated with portal hypertension, frequently jeopardizing the possibility of major hepatectomy due to the associated risk of PHLF. However, functional evaluation has allowed the extension of the pool of patients who could be more safely submitted to liver resection. According to an expert consensus meeting, PVE is usually indicated in cirrhotic patients when the FLR is ≤ 40% of TLV. [98] Estimation of the underlying grade of fibrosis/cirrhosis and portal hypertension may be safely done using transient elastography (ultrasound or MR) avoiding the need for and consequent risks of percutaneous liver biopsy.

#### Expert panel recommendations

- The recommended minimum FLR cutoff volume for upfront major hepatectomy in patients with proven liver cirrhosis is 40% of TLV;
- Liver cirrhosis should be assessed preferably using transient elastography or by other non-invasive methods;
- Liver function assessment should be always performed together with conventional volumetry to assess the risk of PHLF in these patients.

### ***Indications (***Fig. 3***)***

**Fig. 3:**
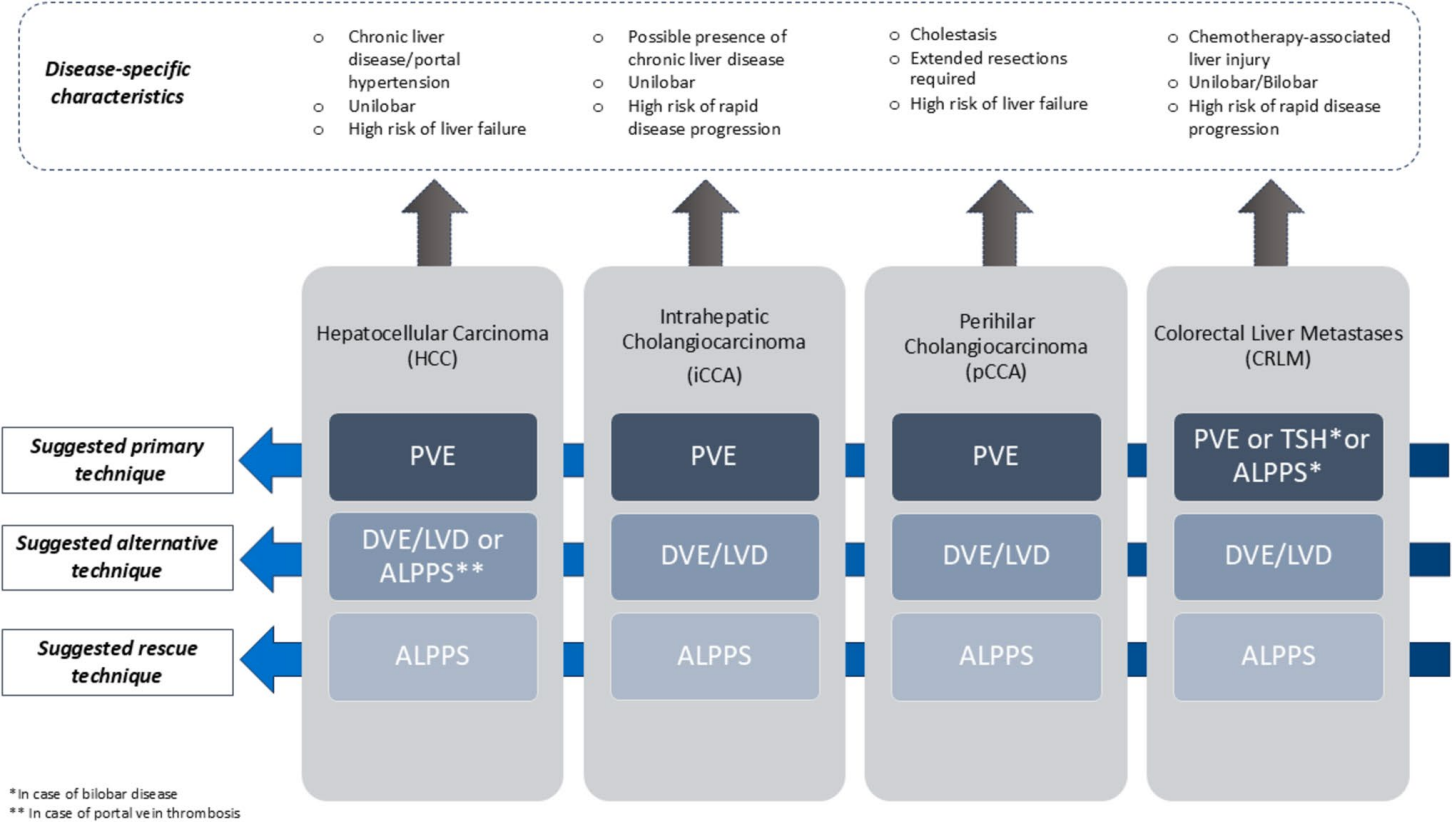
Summary of surgical strategies to enhance future liver remnant according to different indications. ALPPS = Associating Liver Partition and Portal vein Ligation for Staged hepatectomy; DVE = double vein embolization; LVD = liver venous deprivation; PVE = portal vein embolization; TSH = two-stage hepatectomy

#### Which hypertrophy techniques should be considered as the first choice in hepatocellular carcinoma (HCC)?

When searching for studies analyzing different preoperative strategies to increase resectability in HCC, a systematic review and meta-analysis including a total of 411 patients showed that resectability and FLR hypertrophy were higher in ALPPS compared to PVE and radiation lobectomy but at the expense of a higher rate of complications (38%) [99]. However, ALPPS can be considered in cases of portal vein thrombosis or failed PVE [100].

When TACE + PVE was compared to PVE alone, combined loco-regional treatments showed more favorable results in terms of resectability, overall survival, disease-free survival, and percentage increase in FLR. [101]

Data on DVE/LVD in the setting of HCC are still not available with no specific outcomes data in the subgroup of patients with HCC. Possible hemodynamic changes following LVD may include a lower arterial buffer response due to hepatic vein embolization and an increased risk of portal hypertension (a possible role of HVPG gradient measurement to decide for PVE versus LVD may be speculated).

##### Expert panel recommendations

- PVE is the recommended approach for liver hypertrophy in patients with HCC and normal liver function;
- Research on the possible use of combined PVE or DVE/LVD with loco-regional treatments should be encouraged.

#### Which hypertrophy techniques should be considered as the first choice in intra-hepatic cholangiocarcinoma (iCCA)?

Induction of hypertrophy of FLR is needed in the setting of iCCA as major and extended resections are frequently required. Disease progression is the most frequent cause of drop-out, still affecting a significant proportion of patients (up to 38%, as reported by Nevermann et al. [85]). No meta-analyses comparing different hypertrophy techniques in iCCA alone have been published so far; however, PVE is still considered the standard approach in patients with insufficient FLR, resulting in a significant decrease in PHLF and 90-day mortality in patients with iCCA undergoing major liver resection. [102] Despite the International ALPPS registry reported a decreased morbidity (29%) and mortality (7%) and superior overall survival compared to palliative chemotherapy, [103] biliary tumors in general (iCCA and pCCA) have been demonstrated to be a significant risk factor for 90-day mortality in the latest report of the Italian ALPPS Registry. [46]

Outcomes and results of DVE/LVD in iCCA have not been specifically reported yet, but their safety and efficacy have been shown in other indications for liver resection. The advantage of DVE/LVD over ALPPS in iCCA patients could be the avoidance of a surgical procedure (as candidates to hypertrophy techniques generally have unilobar disease) while shortening the waiting time before surgery.

##### Expert panel recommendations.

- PVE is the recommended approach for liver hypertrophy in patients with iCCA.

#### Which hypertrophy techniques should be considered as the first choice in pCCA?

PVE is proven to be safe and effective, increasing the resectability rate in the setting of pCCA and being associated with reduced risk of liver failure and mortality in high-risk resections as reported in the matched cohort of patients who underwent PVE for pCCA and compared with those who did not undergo PVE. [104] In a recent European survey, PVE was indeed the most frequently used technique in the setting of pCCA while most centers still do not have any experience with DVE/LVD [105]. No meta-analyses comparing different hypertrophy techniques in pCCA alone have been published so far.

Early studies and case series have shown that DVE/LVD can induce a better hypertrophy of the FLR in terms of liver function and volume within a shorter time frame compared to PVE. In the case–control study by Marino et al., post-procedural FLR function, calculated using 99 m-Tc-Mebrofenin hepatobiliary scintigraphy, and kinetic growth rate were greater in the DVE/LVD cohort compared to the PVE cohort. [106]

While ALPPS may enable faster FLR growth compared to PVE, mortality data are discouraging. International ALPPS Registry reported a mortality of 48% in 2017 [53] and even though more recent series tend to show better results of ALPSS in pCCA with a 22% mortality rate [58], the use of ALPPS should be discouraged in pCCA. [107]

##### Expert panel recommendations

- PVE is the recommended approach for liver hypertrophy in patients with pCCA
- Data on DVE/LVD are promising but still preliminary; no recommendations can be made at present.

#### Which hypertrophy techniques should be considered as the first choice in patients with colorectal liver metastases?

Portal vein embolization (PVE) is a validated and established technique to increase FLR volume in patients with CRLM and a small FLR [108], [109]. In patients with bilobar CRLM and small FLR, TSH has been the standard approach for several years. After the introduction of ALPPS, preliminary results were characterized by elevated mortality and morbidity rates. Nowadays, these outcomes have been shown to be comparable to TSH with even higher resection rates in favor of ALPPS as suggested not only by the LIGRO trial [36]. [33] However, when looking at two other recent meta-analyses, major complications were higher in the ALPPS group while 90-day mortality was similar between the two groups [34], [35]. Despite the higher rate of resectability in ALPPS which may explain the higher OS in the ALPPS group as shown in the LIGRO trial, [36] the published meta-analyses in this regard did not show any survival advantage of ALPPS over TSH [34], [35].

Data on DVE/LVD specifically in the setting of CRLM as an alternative technique to PVE in staged hepatectomies are promising. The DRAGON 1 trial, which included 102 patients from 42 centers, aimed to evaluate the safety of the procedure, reporting a resectability rate of 90%, severe morbidity of 34%, and mortality of 6%. [27] Two RCTs comparing PVE and DVE/LVD in patients with CRLM are still recruiting and results from these studies are eagerly awaited.

##### Expert panel recommendations

- TSH and ALPPS can both be considered for patients with bilobar CRLM but no recommendation can be made in favor of one procedure over the other. The choice of surgical technique should be left to the surgeon’s discretion and tailored to the case;
- Data on DVE/LVD are promising but still preliminary; no recommendation can be made at present pending the results of ongoing RCTs. Until then, PVE remains the recommended approach for liver hypertrophy.

#### Which hypertrophy technique should be performed in the setting of liver transplantation (RAPID)?

The RAPID technique (Resection And Partial Liver Transplantation With Delayed Total Hepatectomy) has been recently described for patients with nonresectable colorectal liver metastases utilizing left lateral or left liver splits from deceased or living donors [110, 111]. Among all available portal vein occlusion (PVO) techniques that could be used to increase the transplanted liver in the interstage period, PVL has been described as the procedure of choice. Functional imaging tests such as hepatobiliary scintigraphy may have a potential role in RAPID to support the decision to complete the second stage of hepatectomy [110, 112].

##### Expert panel recommendations

- Portal vein ligation is the recommended hypertrophy technique in RAPID setting.

## Discussion

The I GROWtoH position paper highlights the transformative potential of liver hypertrophy techniques in surgical oncology. Deep inconsistencies and lively debate across institutions in treatment strategies, together with the relatively rare use of these techniques, increase the difficulty to generate robust evidence on this topic, hence widening divergence. By advancing the understanding and implementation of these methods, patient outcomes can be enhanced and the horizons of surgical intervention for liver tumors further broadened.

To achieve this, continued research is essential to refine these techniques and develop standardized protocols. In this context, I GROWtoH provides a valuable platform for ideas exchange and working on collaborative projects, going beyond its foundational role in documenting national trends. Its prospective registry structure enables multicenter studies that can offer critical insights into patient selection, procedural efficacy, and long-term outcomes especially for newer techniques such as DVE/LVD. Furthermore, a national registry scenario without previous screening of centers according to activity caseload offers an ideal environment to evaluate the safety of complex surgical strategies in a real-world setting. Additionally, exploring combined approaches (e.g., PVE or DVE/LVD with locoregional therapies) holds potential to enhance the effectiveness of existing liver hypertrophy methods. Emerging pharmacologic adjuncts—most notably small-molecule MKK4 inhibitors, which promote hepatocyte proliferation and dampen stress-kinase signaling—may further increase the speed and safety of postoperative hypertrophy and therefore deserve formal future evaluation alongside each technique [113].

While we recognize that the methodology of this position paper has inherent limitations, particularly the absence of formal grading of evidence and recommendations, we submit that it represents a significant step forward. It reflects the collective effort of the Italian surgical community to build consensus on key aspects of regenerative liver surgery, laying the groundwork for future evidence-based guidelines and ultimately an international consensus.
